# Supplementary figures and images for: Detection of nitric oxide-mediated metabolic effects using real-time extracellular flux analysis
Source: PLoS One. 2024 Mar 7;19(3):e0299294. doi: 10.1371/journal.pone.0299294 (PMC10919732; doi:10.1371/journal.pone.0299294)

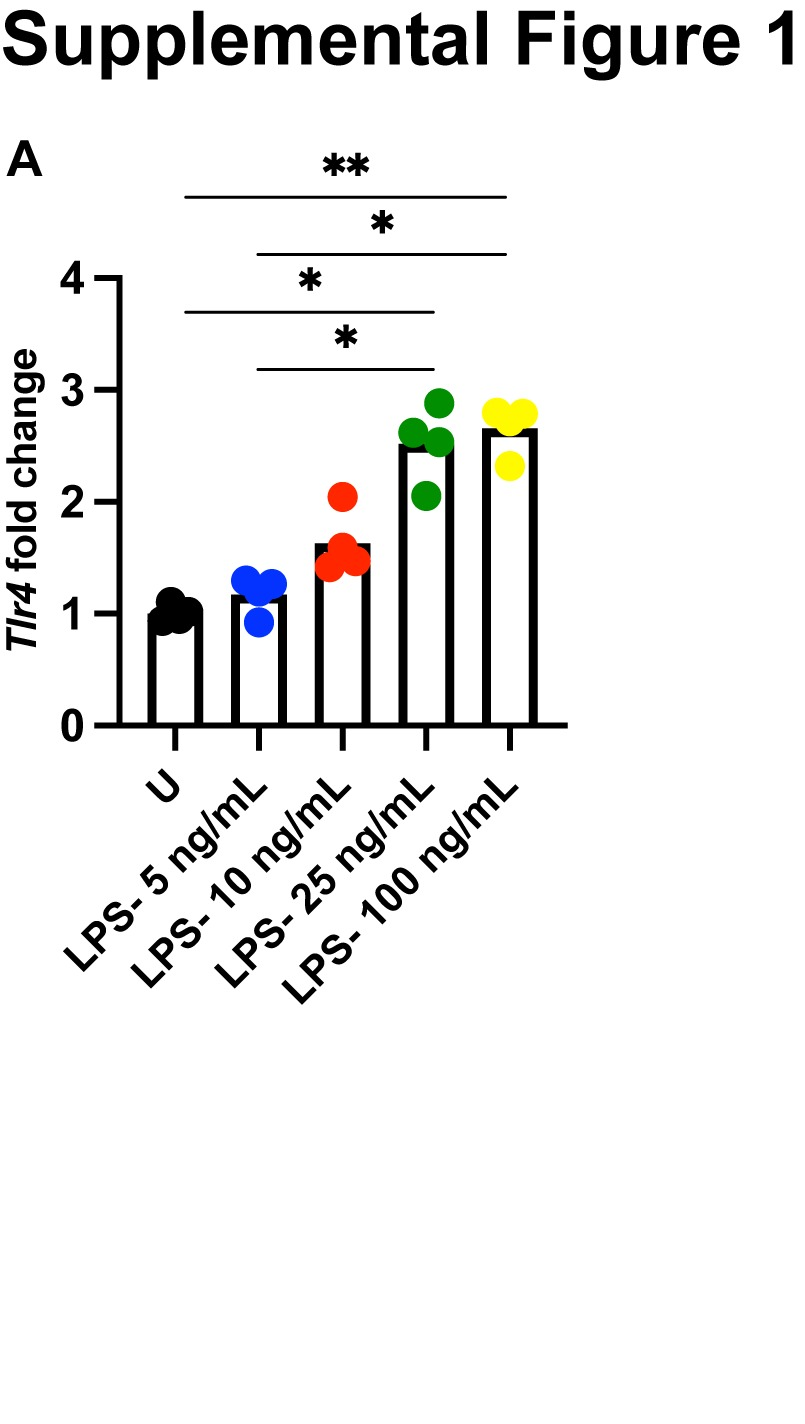

Supplement: S1 Fig — (A) Tlr4 gene transcription assessed by RT-qPCR from BMDCs stimulated for 5 hours with U, 5 ng/mL, 10 ng/mL, or 25 ng/mL LPS. Signals normalized to β-actin as the housekeeping gene via the 2(ΔCt) method. Analyzed by one-way ANOVA, adjusted p values are reported (p value > 0.05 ns), n = 4 biological replicates, representative of at least three independent experiments. * p <0.05, ** p <0.01, *** p <0.001, **** p <0.0001. (TIF) [file pone.0299294.s001.tif]

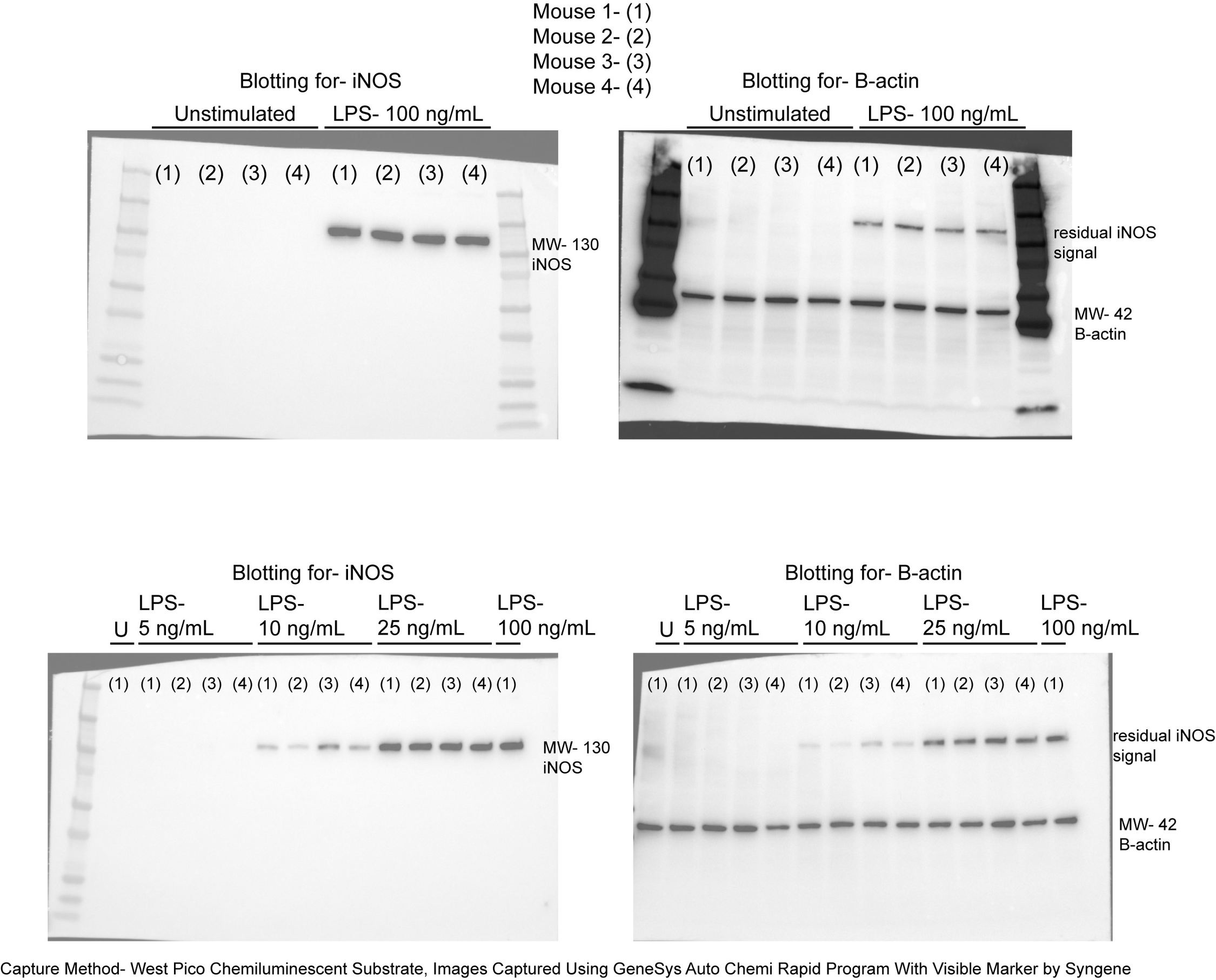

Supplement: S1 Raw images — (TIF) [file pone.0299294.s003.tif]
