# Supplementary material for: Detection of nitric oxide-mediated metabolic effects using real-time extracellular flux analysis
Source: PLoS One. 2024 Mar 7;19(3):e0299294. doi: 10.1371/journal.pone.0299294 (PMC10919732; doi:10.1371/journal.pone.0299294)
Supplement: S1 Protocol — (PDF) [file pone.0299294.s002.pdf]

S1 Protocol. Extended materials and procedure for all extracellular flux analysis assays: modified mitochondrial stress test, iNOS inhibitor kinetic assay, and glycolytic burst assay.

1. **Materials**

I. Standard Seahorse Extracellular Flux Analysis

1. Seahorse XFe96 Cell Culture Microplates (Agilent)
2. Seahorse XFe96 Sensor Cartridges (Agilent)
3. Cell culture grade H2O (Corning); This can be substituted with other brands.
4. Complete DC medium (RPMI 1640 containing 10% FBS, 2 mM L-glutamine, 100 Units/ml penicillin, 100 mg/ml streptomycin, and 55 mM 2-βME, all from Life Technologies)
5. Seahorse XF Calibrant (Agilent)
6. Seahorse XF Unbuffered RPMI (Agilent)
7. Fetal Bovine Serum (FBS)
8. D-Glucose 500 mM
9. Seahorse XF Drug Loading Guide (Agilent)
10. 96-well flat bottom plate(s) (optional)
11. Multi-channel pipette and reservoir (optional)
12. 15 mL and 50 mL conicals (optional)
13. 37°C CO_2 Incubator for overnight stimulation with LPS
14. 37°C Non-CO_2 Incubator for after run media is added.

II. Seahorse Mitochondrial Stress Test

1. All materials from (I.)
2. LPS-EB Ultrapure (InvivoGen); This can be substituted with regular LPS or other TLR agonists.
3. Complete DC medium
4. Standard Mitochondrial Stress Test Drugs (bought separately)
    a. Oligomycin 1 μM (Thermo Scientific)
    b. FCCP 1.5 μM (Cayman Chemical)
    c. Rotenone 100 nM (MP Biochemicals)
    d. Antimycin A 1 μM (Sigma Aldrich)
5. S-ethyl Isothiourea (SEITU) inducible nitric oxide synthase inhibitor 500 mM stock (Cayman Chemical)
6. 1400W inducible nitric oxide synthase inhibitor 125 mM stock (Cayman Chemical)

III. Glycolytic Burst Assay

1. All materials from (I.) minus 37°C CO_2 Incubator
2. LPS-EB Ultrapure (InvivoGen); This can be substituted with regular LPS or other TLR agonists.
3. Complete DC medium
4. S-ethyl Isothiourea (SEITU) inducible nitric oxide synthase inhibitor (Cayman Chemical)

IV. iNOS Inhibitor Kinetic Assay

1. All materials from (I.)
2. LPS-EB Ultrapure (InvivoGen); This can be substituted with regular LPS or other TLR agonists.
3. Complete DC medium
4. S-ethyl Isothiourea (SEITU) inducible nitric oxide synthase inhibitor (Cayman Chemical)
5. 1400W inducible nitric oxide synthase inhibitor (Cayman Chemical)

2. Procedure

This protocol uses BMDCs as the experimental model for activation-associated induction of iNOS and subsequent measurement of NO-mediated effects on cellular respiration.

I. Cell Harvest and Preparation

1. BMDCs are generated according to our recent publication [6]. Briefly, femurs are gathered from ~8-week-old C57BL/6J mice. Bone marrow cells are flushed, resuspended, and cultured in complete DC medium (CDCM) containing RPMI 1640 supplemented with 10% FBS, 2 mM L-glutamine, 100 Units/ml penicillin, 100 mg/ml streptomycin, 55 mM 2-βME, plus 20 ng/mL murine recombinant GM-CSF for 7 days with a media exchange every other day.
2. 7-day GM-CSF differentiated BMDCs are harvested and diluted to $2x10^6$ in CDCM not containing GM-CSF.
3. BMDCs are seeded in the XF Cell Culture Microplate at 200,000 cells per well in the plate according to parameters needed. To do this properly, follow the Adherent Cells in Agilent Seahorse XF96 Tissue Culture Microplates User Guide (Agilent). Briefly, add 100 μL of $2x10^6$/mL BMDCs at a ~45-degree angle where the end of the pipette tip sits around halfway down the side wall of the well touching the wall itself. When cell suspension is dispensed, the end of the pipette tip should be just below the top of the liquid.
4. Place the XF Cell Culture Microplate into a 37°C incubator in 5% CO_2 while you prepare your treatment reagents for the different assay types. BMDCs need time to adhere and settle to the bottom of the plate. Leave them in the incubator for a minimum of 30 minutes before treatment.
5. If using cell types that differ from BMDCs or other myeloid-derived cells such as macrophages, you may have to adjust the seeding density to ensure a confluent monolayer in the XF Cell Culture Microplate. Additionally, cell types that are adherent versus partially adherent or non-adherent may require coating of the XF Cell Culture Microplate to ensure proper monolayer conditions.

II. Seahorse Mitochondrial Stress Test

1. The Seahorse Mitochondrial Stress Test is performed as standardly done in our recent publication, however there are some major differences discussed below. Briefly, BMDCs are treated with various concentrations of LPS and set in the

37°C CO_2 Incubator for overnight stimulation. Don't forget to hydrate the Seahorse XFe96 Sensor Cartridge with 200 μL of ccH_2O per well.

2. Around 18-24 hours post activation, cells are taken out of the 37°C CO_2 Incubator and centrifuged for 5 minutes at 1500 rpm. While centrifuging, replace the ccH_2O in the Seahorse XFe96 Sensor Cartridge with 200 μL of XF Calibrant.
3. After the spin, ~200 μL cell supernatant is carefully taken off the top of the cell monolayer using a multichannel pipette and transferred to a 96-well flat bottom plate for use in downstream applications such as the Griess nitrite assay for indirect nitric oxide quantification.
4. XF Run Media is made by combining 47 mL XF RPMI, 2.5 mL FBS, and 0.5 mL 500 mM glucose and 180 μL is added to each well by multichannel. Cells are set in the 37°C Non-CO_2 Incubator to acclimate.
5. 10X Seahorse Mitochondrial Stress Test drugs are made by adding 40 μL drug to 4 mL of plain XF RPMI. There will be a total of 4, 15 mL conical, one for each drug (Oligomycin, FCCP, and Rotenone/Antimycin A) plus one for an iNOS inhibitor (either SEITU or 1400W).
6. Using the drug loading guide, the Seahorse Mitochondrial Stress Test drugs are loaded into their respective drug ports (A-Oligomycin, B-FCCP, C-Rotenone/Antimycin A, D-iNOS inhibitor) within the Seahorse XFe96 Sensor Cartridge. To do this, the multichannel pipette is set to 25 μL and the drugs are dispensed only to the first stop, which should be ~20 μL.
7. Once drugs are loaded into the Seahorse XFe96 Sensor Cartridge, it is time to program the machine to perform the assay.
8. On Wave, open a blank program. In the group definition tab, add 1 injection strategy, add drug for each port and designate the ports with the drugs specified in step 6.
9. Proceed to enter in treatments, assay medias, and other labeling identifiers for intended experiment. Generate groups. Proceed to the plate map tab.
10. In the plate map tab, assign a color identifier to the well in which the experimental condition will be in on the plate layout. Proceed to the assay protocol tab.
11. In the protocol tab, our Mitochondrial Stress Test includes 3 baseline measurement cycles (3-minute mix, 3-minute read). Add 4 injections. These should populate in sequential addition starting with an injection for port A to port D. Each injection has 3 measurement cycles (3-minute mix, 3-minute read). If planning on using SEITU as the fourth injection iNOS inhibitor, 3 measurement cycles is enough to see the desired effect. If planning to use 1400W instead, extend the measurement period 50-100 minutes past the injection to see the entire intended effect. The assay should say it takes 1:42-1:45 to complete. Proceed to run assay if all specifications are correct.
12. In the run assay tab, designate a file name. Copy the file name. Click run assay when Seahorse XFe96 Sensor Cartridge has all drugs loaded. Paste the file name to designated file destination and click save. Once saved, the machine will open the plate loader.
13. Add the Seahorse XFe96 Sensor Cartridge in the correct orientation (Well A1 at top left). Make sure to take the lid off. Ensure that drugs are loaded in all ports, and then tell Wave to load the plate.

14. The calibration period will take approximately 20-25 minutes to complete. At the end of the period the machine light will turn from blue to yellow to signify that it is ready to load the XF Cell Culture Microplate. Ensure that all the oxygen and hydrogen ion calibrations are green checkmarks before proceeding. If they are not, the plate was not properly hydrated (this could be for several reasons such as not being left to hydrate for long enough, or not swapping from ccH_2O to calibrant before running). If all green checkmarks, click load cell plate. Take XF Cell Culture Microplate from non-CO_2 incubator and load onto the machine again in proper orientation (Well A1 at top left). Take off lid and click load plate.
15. The assay will initialize, run equilibrium measurements, and begin baseline measurements. This assay after calibration should take ~2 hours to complete.
16. Once assay is complete, take out the Seahorse XFe96 Sensor Cartridge and XF Cell Culture Microplate from the machine. Click see results.
17. In the results tab, click export and choose from the file formats available. We primarily work with prism files; however, Excel files work fine as well.

III. <u>iNOS Inhibitor Kinetic Assay</u>

1. This assay is a hybrid of the standard Mitochondrial Stress Test and the Glycolytic Burst Assay where cells are stimulated overnight, and the assay is run for a longer period (~6-8 hours).
2. BMDCs are prepared as stated above by loading only in the middle of the plate, then treated with various concentrations of LPS and set in the 37°C CO_2 Incubator for overnight stimulation. Hydrate the Seahorse XFe96 Sensor Cartridge with ccH_2O overnight before running this assay.
3. Around 18-24 hours post activation, take the cells out of the 37°C CO_2 Incubator and centrifuge for 5 minutes at 1500 rpm. While centrifuging, replace the ccH_2O in the Seahorse XFe96 Sensor Cartridge with 200 μL of XF Calibrant.
4. Post centrifuging, collect cell supernatant and then prepare XF Run Media (both detailed above in II.3 & II.4).
5. Since the Seahorse XFe96 machine is not humidified, two rings of wells around the middle of the plate will get 200 μL of ccH_2O to act as a micro humidifier. Make sure to add 180 μL of XF Run Media to the four corner wells, instead of ccH_2O, which are used during the calibration period. Place the cell plate into the 37°C Non-CO_2 Incubator to acclimate.
6. To ensure proper control of all variables, we prepared the plate with both SEITU and 1400W as a part of the XF Run Media and as 10X injections. Since the effect of SEITU as a component of the Run Media is well documented in the Glycolytic Burst Assay, we have only detailed the iNOS inhibitors as 10X injections drugs here, however they were included in the original assay for quality control.
7. 10X SEITU is made by adding 20 μL of 500 mM SEITU to 2 mL of plain XF RPMI.
8. 10X 1400W is made by adding 40 μL of 250 mM 1400W to 2 mL of plain XF RPMI.

9. Using the drug loading guide, 10X iNOS inhibitors are loaded into drug port A of the respective SEITU or 1400W-assigned wells while all other wells receive plain XF RPMI.
10. To program the iNOS Inhibitor Kinetic Assay onto Wave, open a blank program. Add a Baseline cycle with 6 reads. Add an injection for port A which is for the iNOS inhibitors. Ask the machine to measure for 20 cycles after injecting (standard 3 minutes mix and 3 minutes measure). Add a measurement period after the injection period for another 60 measurement cycles. Together, this assay should have a total run time of 8 hours.
11. Place the Seahorse XFe96 Sensor Cartridge into the machine and perform the calibration then the assay itself after loading the cell plate. The assay should take ~8 hours to finish plus ~1 hour of preparation.

IV. Glycolytic Burst Assay

1. Unlike the standard Mitochondrial Stress Test, the Glycolytic Burst Assay is performed in one day and is performed over a longer period (10 to 12 hours). The Seahorse XFe96 Sensor Cartridge needs to be hydrated with XF Calibrant at least 3-4 hours before running this assay (like the Mitochondrial Stress Test you can hydrate the day before running as described above in II.1).
2. Cells are prepared as stated above, however, cells should only be loading in the middle of the plate.
3. After letting the cells settle and adhere in the 37°C CO_2 Incubator for at least 30 minutes, cells should be taken out and centrifuged for 5 minutes at 1500 rpm. CDCM should be dumped and replaced with 180 μL of XF Run Media (made above in II.4). Half the plate should receive plain XF Run Media, and half the plate should receive XF Run Media (6 μL 500 mM SEITU + 6 mL XF Run Media).
4. Since the Seahorse XFe96 machine is not humidified, two rings of wells around the middle of the plate will get 200 μL of ccH_2O to act as a micro humidifier. Make sure to add 180 μL of XF Run Media to the four corner wells, instead of ccH_2O, which are used during the calibration period. Place the cell plate into the 37°C Non-CO_2 Incubator to acclimate.
5. 10X LPS is made by adding 40 μL of 100 μg/mL LPS to 4 mL of plain XF RPMI.
6. Using the drug loading guide, 10X LPS is loaded into drug port A of the respective LPS-assigned wells while all other wells receive plain XF RPMI.
7. To program the Glycolytic Burst Assay onto Wave, open a blank program. Add a Baseline cycle with 6 reads. Add an injection for port A which is for LPS. Ask the machine to measure for 20 cycles after injecting (standard 3 minutes mix and 3 minutes measure). Add a measurement period after the injection period for another 92 measurement cycles. Together, this assay should have a total run time of 12 hours.
8. Place the Seahorse XFe96 Sensor Cartridge into the machine and perform the calibration then the assay itself after loading the cell plate. The assay should take ~12 hours to finish plus ~1 hour of preparation.
